# Deformation-Induced Martensitic Transformation in Laser Cladded 304 Stainless Steel Coatings

**DOI:** 10.3390/ma15186392

**Published:** 2022-09-14

**Authors:** André T. Zeuner, Leonid Gerdt, Andrea Ostwald, Peter Grün, Maria Barbosa, Jörg Kaspar, Martina Zimmermann

**Affiliations:** Fraunhofer Institute for Material and Beam Technology IWS, 01277 Dresden, Germany

**Keywords:** laser cladding, deformation-induced martensitic transformation, wear and corrosion resistance, 3-point bending

## Abstract

There are only a few cost-effective solutions for coating applications in combined mechanical loading and corrosive environments. Stainless steel AISI 304 has the potential to fill this niche, showing excellent corrosion resistance while utilizing the deformation-induced phase transformation from γ-austenite to α’-martensite, which results in an increase in strength. However, it is not known whether this can occur in laser cladded material. Therefore, laser cladded AISI 304 coatings in as-cladded condition and after heat treatment at 1100 °C for 60 min were investigated before and after bending deformation, by means of light microscopy, energy-dispersive X-ray spectroscopy and electron backscatter diffraction. It was shown that due to the dendritic microstructure accompanied by an inhomogeneous distribution of the main alloying elements (Cr and Ni), no deformation-induced phase transformation occurred in the as-cladded coating. The applied approach with subsequent solution heat treatment at 1100 °C for 60 min resulted in a homogeneous γ-austenite microstructure, so that a deformation-induced martensitic transformation (DIMT) could occur in the coatings. However, the volume fraction of martensite that had been formed locally at individual shear bands was rather low, which can be possibly attributed to the high Ni content of the feedstock, stabilizing the γ-austenite microstructure. This study shows the possibility of exploiting the DIMT mechanism in heat-treated laser-cladded AISI 304 coatings.

## 1. Introduction

Laser cladding (LC) is increasingly used in industrial applications, such as mechanical and plant engineering, vehicle construction or aerospace [1], for the production of high-quality corrosion and wear protection coatings. In LC, a melt pool is created by a laser beam focused on the substrate surface, and the coating material is added in the form of powder or wire. The coating material melts completely and solidifies as a dense layer with a metallurgical bond to the substrate. The laser beam and respective optics configuration offer the possibility to control the energy input, intensity distribution, and the melt pool size very precisely. Thus, high-quality coatings with high final contour accuracy, low change in chemical composition, and very good reproducibility can be manufactured. Furthermore, the solidification process and the microstructure formation can also be specifically influenced by the process parameters that are chosen for the cooling phase [2].

A broad range of coating materials are used, depending on the application. For wear and corrosion protection, high coating quality and performance can be achieved with Ni- and Co-base alloys [3,4]. However, with the increasing shortage of raw materials, availability is decreasing and prices are raising [5]. Furthermore, Ni- and Co-based alloys are partly classified as harmful to health. This results in a high economic and ecologic motivation to substitute these alloys [6]. As a result, Fe-based alloys represent a promising alternative, although significant compromises have to be accepted, for example, in terms of their wear and corrosion behavior. To protect printing press rollers from corrosion, for example, the stable austenitic stainless steel AISI 316 is used. This alloy has excellent corrosion properties due to high chromium contents, added molybdenum and its face-centered cubic crystal structure [7]. However, AISI 316 exhibits low hardness and yield strength and thus very limited wear resistance. This limits the application fields of this alloy as a coating material. Studies to improve wear resistance—for example by surface hardening [8]—were partly successful but did not lead to widespread substitution of Ni- and Co-based coating systems.

Stainless steel AISI 304, on the other hand, is known to have good corrosion resistance with comparatively high mechanical properties. These properties can be attributed to a deformation-induced phase transformation of γ-austenite to α’-martensite [9]. This phenomenon has already been studied in great detail on sheet metal. It was shown that the transformation behavior depends on the stacking fault energy of the material [10,11], which in turn is influenced by chemical composition [12], ambient temperature [13], and deformation parameters [14]. Compared to γ-austenite, α’-martensite shows a significantly higher hardness, strength, and the change in lattice structure during γ→α’ transformation is associated with a volume increase. The latter leads to the development of residual compressive stresses in the material, which can hinder or even completely stop crack growth [15]. The behavior of the cold rolled AISI 304 under wear condition has been recently investigated by Mao et al. [16]. The authors investigated the friction-induced martensitic transformation and showed its dependence on the austenitic grain size. Although the phase transformation behavior has been well studied on bulk material and AISI 304 has been successfully processed by LC [17,18,19], to the authors’ knowledge the transformation behavior of AISI 304 coatings synthesized by LC has not been studied so far. AISI 304 coatings provide a far broader potential for applications with high mechanical loadings than other stainless steels, such as AISI 316, due to the ability for deformation-induced martensitic transformation. Furthermore, AISI 304 benefits from a less cost-intensive chemical composition compared to Ni- or Co-based coating materials. However, the process-structure-property relationships must first be investigated and the influencing variables on the martensitic transformation evaluated. This work, therefore, investigates the prerequisites that must be fulfilled for a LC coating of AISI 304 to show a DIMT.

## 2. Materials and Methods

The chemical composition of the substrate steel DD13 and steel AISI 304 powder used in the current study is given in Table 1. The experimental procedure includes the coating deposition by means of laser cladding, characterization of the coating’s microstructure, and the evaluation of deformation behavior under bending load by means of 3-point bending tests. The AISI 304 powder (NDM New Materials Development GmbH, Germany) had a particle size distribution between 45 and 90 µm.

**Figure 1:**
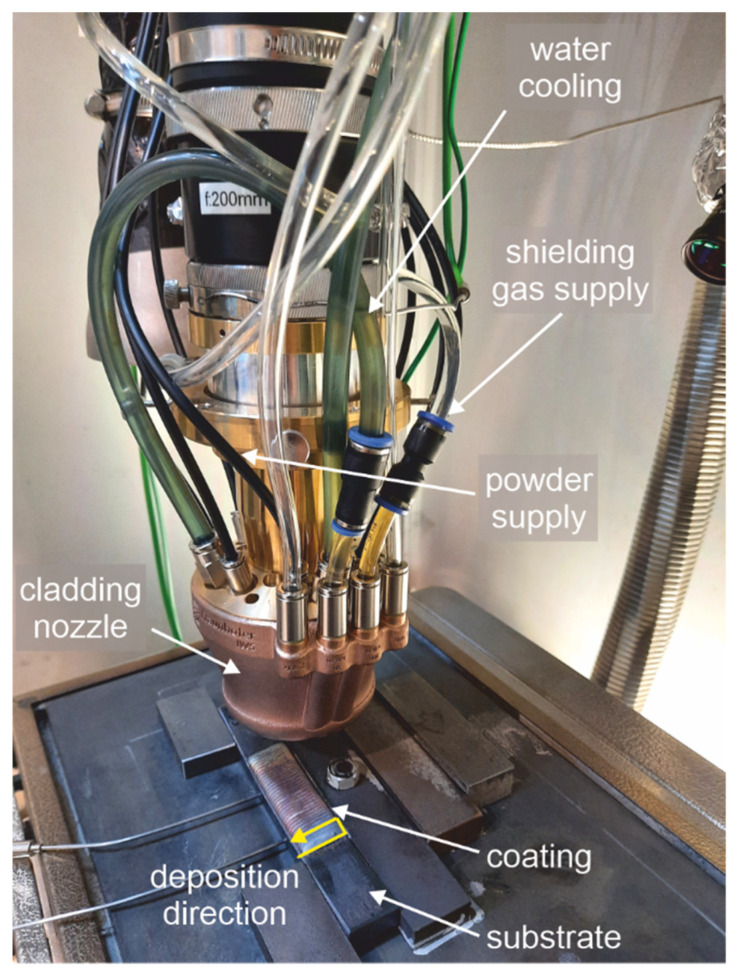
The test assembly with the sample after laser cladding process.

The laser cladding tests were carried out using the cladding nozzle COAXpowerline developed at the Fraunhofer IWS [20]. This is a coaxial ring nozzle with a powder focus diameter of 1.9 to 2.2 mm. The size of the powder focus diameter is determined by the powder feed rate and its carrier gas volume flow. Laser cladding took place in an argon atmosphere at a working distance (distance between substrate and nozzle tip) of 13 mm. A laser spot diameter of 3.81 mm was used for optimum powder utilization. A 4 kW diode laser from Laserline GmbH (Mülheim-Kärlich, Germany) was used for the laser cladding process. The test assembly can be seen in Figure 1. The total powder feed rate was 10 g/min. All samples were welded with a laser power of 1700 W and a process velocity of 700 mm/min. The total number of deposited tracks in one layer was set to 34 with a spacing between the tracks of 1.8 mm. The coating comprised of 3 layers with an overall thickness of about 3 mm.

A part of the generated coatings were examined without further post-treatment and will be referred to as “as-cladded” in the rest of the paper. The other part was subjected to heat treatment, in order to homogenize the microstructure and to achieve a fully austenitic state. For this purpose, the samples were solution annealed in an oven flushed with argon gas at 1100 °C for one hour. Subsequently, they were rapidly cooled by means of compressed air. The heat treatment was carried out in order to compare the as-cladded coatings with a condition more comparable to that of hot-rolled and annealed sheet material, on which the phase transformation behavior has already been intensively studied [21]. The annealed condition will be referred to as “heat-treated” in the rest of the paper.

**Figure 2:**
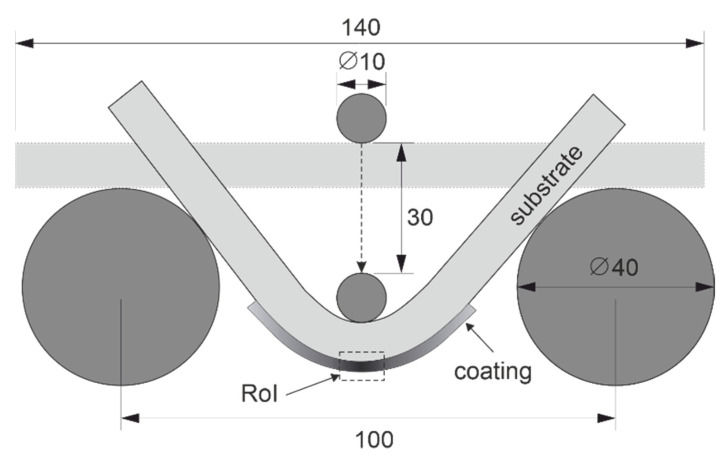
Experimental set up of 3-point bending test, dimensions in mm.

In order to examine the possibility of deformation-induced martensitic transformation in the laser cladding coatings of AISI 304 steel, 3-point bending tests were conducted. This technique enables a clearly defined localization of the maximum deformation in the loaded sample. The maximum elongation occurs at the midpoint of the samples under the applied force. Here, the region of interest (RoI) was specified in order to investigate the deformation behavior of the AISI 304 coating (Figure 2).

The maximum displacement of the middle stamp was set to 30 mm. According to Equation (1), the calculated maximum flexural strain (ε_f_) in the outer surface of the coating at the maximum deflection is about 20%.

(1) $$\varepsilon_{f}=\frac{6\mathrm{Dd}}{L^{2}}$$

where d is the thickness of the tested sample and L is the distance between the outer support rollers.

The microstructural evolution in the RoI were investigated on prepared cross sections of the samples after the bending tests. The samples were ground with SiC abrasive paper, wet-polished using a diamond polishing suspension, and finally vibratory polished for multiple hours using Al and Si oxide polishing suspensions. This elaborate preparation was carried out because AISI 304 can show phase transformation during overly intensive mechanical processing. The preparation-induced α’-martensite formation would falsify the analyses. The samples for light microscopy were additionally etched with V2A etchant containing 100 mL HCl, 100 mL H_2_0 and 10 mL HNO_3_. The metallographic analysis was performed using a scanning electron microscope (SEM) (JEOL JSM-7800F, Akishima, Japan), energy dispersive X-ray spectroscopy (EDS) device Oxford X-Max, and the NordlysNano EBSD detector (Abingdon, UK) for the electron backscattered diffraction, together with a light microscope Olympus GX51 (Tokyo, Japan). EBSD scans were performed on the samples tilted by 70° with a step size of 0.15 µm and the acquired data was analyzed with AZtecCrystal 2.1 software (Oxford Instruments, Abingdon, UK).

## 3. Results and Discussion

**Figure 3:**
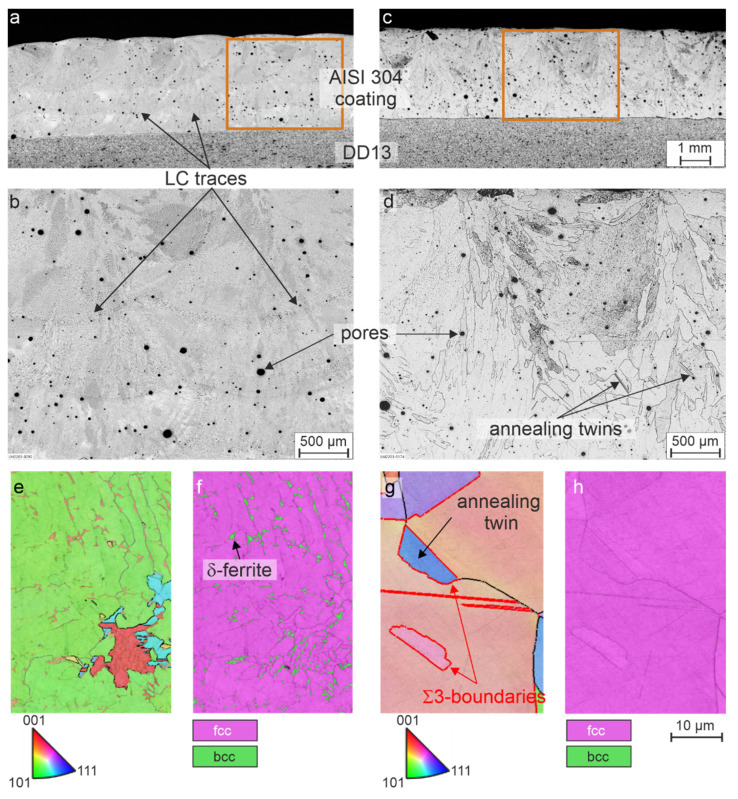
Cross sections of (**a**) coating of AISI 304 in as-cladded condition with (**b**) higher magnification image, and (**c**) after heat treatment at 1100 °C for 60 min with (**d**) higher magnification image; (**e**) EBSD orientation map of the coating in as-cladded condition with (**f**) corresponding phase map and (**g**) EBSD orientation map of the coating after heat treatment at 1100 °C for 60 min with (**h**) corresponding phase map.

The findings made by light microscopy of the coatings without plastic deformation are presented first. Figure 3a shows a metallographic cross-section of the as-cladded coating, and Figure 3b shows a higher magnified image of the microstructure. In these images, the tracks and overlapping typical of LC are clearly visible, as well as a considerable porosity. The high porosity is a disadvantageous mechanical property for the material as a coating in a possible application and can be considerably reduced by optimization of the powder or process parameters, but is irrelevant for the phase transformation behavior, which is the focus of the current study. It can also be seen that the microstructure is very inhomogeneous, due to manufacturing the coating layer by layer and, consequently, re-heating/re-melting of the lower layers during the deposition of the upper ones. The predominantly dendritic microstructure of the laser cladded AISI 304 can be recognized from the images in Figure 3a,b, and will be examined in detail in subsequent investigations by EBSD and EDS. The EBSD phase map in Figure 3f shows that the microstructure of the LC coating consist of γ-austenite (fcc) and δ-ferrite (bcc). In Figure 3c,d, the cross section of a heat-treated coating is shown in a similar way. As a result of the heat treatment at 1,100 °C for 60 min, the LC traces are no longer visible due to homogenization resulting from the solution treatment, and the microstructure can be described as coarse-grained, even though the grains that are observable show considerable differences in their size. In addition, twins are recognizable. EBSD orientation map (Figure 3g) shows exemplary annealing twins with the typical Σ3 boundaries. Considering the high annealing temperatures and the long holding time, together with the observations made by light microscopy analysis, a fully austenitic microstructure can be assumed. It can be also approved by EBSD analysis of the annealed coating (Figure 3h).

**Figure 4:**
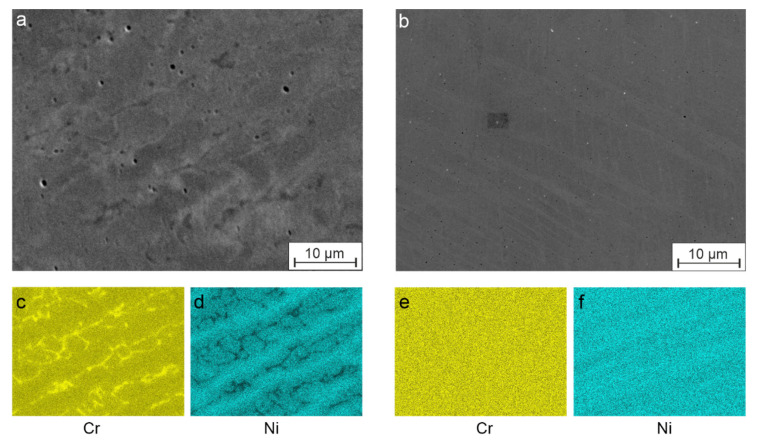
SEM-EDX mappings of the coating AISI 304 for alloying elements Cr and Ni in (**a**) as-cladded condition with elemental distribution of (**c**) Cr and (**d**) Nias well as (**b**) after heat treatment at 1100 °C for 60 min with elemental distribution of (**e**) Cr and (**f**) Ni.

The examination of the coating after cladding and after subsequent heat treatment by means of SEM-EDS is shown in Figure 4. Here, Figure 4a,b show the secondary electron images, and Figure 4c–f show the EDS overlay images for the analysis of the elemental distribution of the main alloying elements (Cr and Ni). Based on these element mappings, the dendritic structure can be recognized, and it can be seen that there was a Cr-rich, Ni-depleted, and in comparison, a lower Cr, Ni-rich region, Figure 4c,d. This constellation indicates δ-ferrite for the Cr-rich region, since it shows a very low solubility for Ni [22]. Accordingly, the second phase should be γ-austenite enriched with Ni, which is stabilized by the high Ni contents. In contrast, the heat-treated coating, which is shown in a similar way in Figure 4e,f, showed an almost homogeneous distribution of Cr and Ni in the element mappings. Accordingly, it was expected to be a metastable γ-austenite phase characteristic of homogenized AISI 304. The austenitic stainless steels generally contain some amount of δ-ferrite in its as-cast microstructure. This is due to the primary ferrite solidification, followed by diffusion-controlled transformation to austenite [23]. Due to the high cooling rates and thus hampered diffusion during laser cladding, a complete δ → γ transformation is almost impossible.

**Figure 5:**
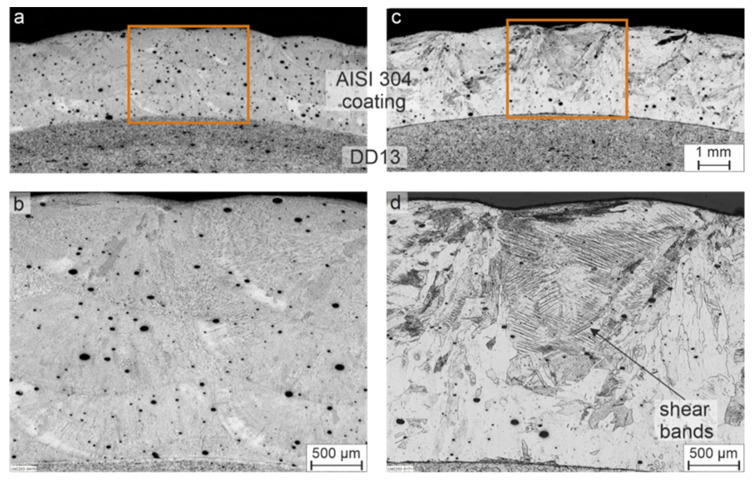
Cross sections of the samples after 3-point bending tests for (**a**) coating of AISI 304 in as-cladded condition with (**b**) higher magnification image, and (**c**) after heat treatment at 1100 °C for 60 min with (**d**) higher magnification image.

Based on the results of the optical microscopy and EDS analyses of non-deformed coatings, it can be assumed that the as-cladded coating is very unlikely to show deformation-induced phase transformation, since neither δ-ferrite nor stabilized γ-austenite are able to transform to α’-martensite. Furthermore, the metallographic cross-section of the as-cladded coating plastically deformed by means of a 3-point bending test in Figure 5a,b showed no evidence of a phase transformation.

In contrast, for the heat-treated coating in Figure 5c,d, the dark etched shear bands can be clearly recognized. Various studies have shown that the formation of α’-martensite occurs preferentially at shear bands or their intersections [24,25]. In the case of the heat-treated coating, a homogeneous elemental distribution of Cr and Ni, indicating metastable γ-austenite, and the formation of shear bands provided the prerequisite for α’-martensite transformation. However, a final interpretation could only be made with a detailed analysis by means of EBSD.

**Figure 6:**
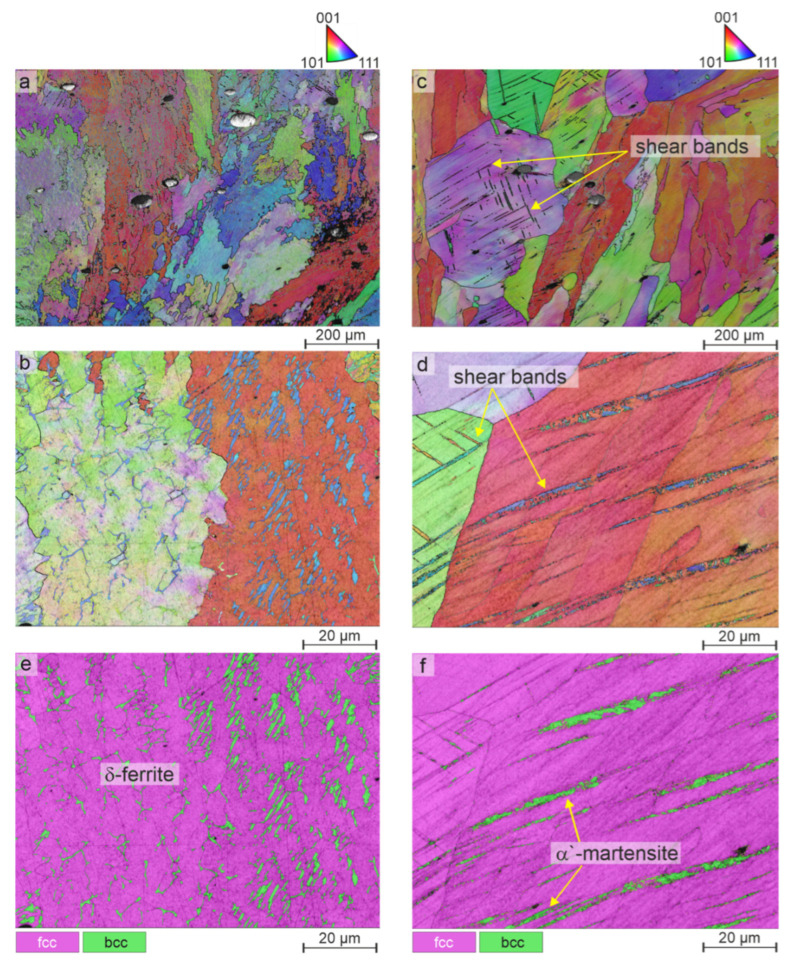
SEM-EBSD orientation maps of (**a**) coating of AISI 304 in as-cladded condition with (**b**) higher magnification image, (**c**) coating of AISI 304 after heat treatment at 1100 °C for 60 min with (**d**) higher magnification image; (**e**) phase map in as-cladded condition and (**f**) phase map after heat treatment at 1100 °C for 60 min.

The results of the EBSD analysis are shown in Figure 6, where Figure 6a is an EBSD orientation map of the deformed as-cladded coating and Figure 6b is a higher magnification image is shown. A phase analysis was carried out on this section (Figure 6c) and it was found that a bcc phase (green) was embedded in the fcc matrix (magenta).

The dendritic morphology, which is very clearly recognizable here, in combination with the bcc structure proved that this was indeed δ-ferrite. Examination of the deformed heat-treated coating in a manner analogous to the as-cladded coating (orientation mappings, Figure 6a–d) and as phase analysis (Figure 6e,f) showed that a bcc phase was present in the shear bands, which is indicative of α’-martensite formation. Although no quantitative analysis of α’-martensite transformation was carried out in these studies, it is apparent that the degree of transformation in the cladded coating is rather low in comparison to what was observed in past studies on bulk material [26,27,28]. This could be attributed to the high Ni content of over 10%, which was listed in Table 1, which stabilized the γ-austenite and thus reduced the driving force for the phase transformation. From these findings, it can be summarized that due to the formation of δ-ferrite and the associated enrichment of the γ-austenite matrix with Ni, an as-cladded coating of AISI 304 does not undergo phase transformation into α’-martensite under plastic deformation. This is problematic for an application, because for pure corrosion protection, AISI 316 is the material of choice due to a higher corrosion resistance. AISI 304 should fill the application field of combined mechanical stress and corrosion, but it shows better mechanical behavior than AISI 316 only under phase transformation. The investigations have shown, however, that this phase transformation only occurs in a homogenized coating. Even in the heat-treated condition, the high Ni content present in this samples partly suppresses the γ to α’ phase transformation. As a consequence, further investigations should be conducted, both regarding process and material, to enable the phase transformation of AISI 304, if possible without post treatment, in order to serve the target application area in a practical way. One possible way to reduce the volume fraction of retained δ-ferrite is to reduce the cooling rate of the coating, for example, by applying an induction heating in order to hold the sample at a moderate temperature subsequent to the cladding process. The effect of the cooling rate on the dissolution of δ-ferrite was investigated by Zargar et al., showing the potential of controlling the volume fraction of δ-ferrite [29].

## 4. Conclusions

As-cladded and heat-treated LC coatings of AISI 304 were investigated for their microstructure and ability to exhibit deformation-induced phase transformation from γ-austenite to α’-martensite. For this purpose, the response of the coatings to the bending load in as-cladded and annealed conditions was determined by means of 3-point bending tests and subsequently examined by metallographic preparation, EDS and EBSD analysis. The findings can be summarized as follows:

1. As-cladded coatings show a dendritic microstructure with a pronounced inhomogeneous distribution of the main alloying elements (Cr and Ni) and a phase composition of δ-ferrite and stabilized γ-austenite.
2. Solution annealing at 1100 °C for 60 min caused an almost homogeneous distribution of the alloying elements in the coating material and led to the formation of a metastable γ-austenite phase.
3. As-cladded coatings did not show any deformation-induced phase transformation due to the presence of δ-ferrite and, therefore, the lack of metastable γ-austenite phase. The presence of δ-ferrite can be explained by segregations of Cr resulting from high cooling rates after deposition process (Figure 4).
4. Due to the generally high Ni contents of the coatings in this work, α’-martensite transformation was observed only locally in individual shear bands of the heat-treated coatings.
5. In order to use AISI 304 LC coatings for an application of combined mechanical and corrosive stress, further research must be carried out on both the process and the material to ensure that pronounced phase transformation can occur, at best in the as-cladded state.

In summary, the response of the LC coatings of AISI 316 steel to bending deformation in terms of DIMT is affected by fast cooling rates in the as-cladded condition. Furthermore, the deviation of the chemical composition of the feedstock, especially regarding increased Ni content, results in stabilization of γ-austenite in the annealed state. Thus, in order to maximize the exploitation of DIMT and consequently increase the damage tolerance of the coatings, further adjustment of chemical composition as well as process optimization should be further investigated. Furthermore, the corrosion and wear resistance of the coatings should be investigated.

## Figures and Tables

**Table 1:** Chemical composition of the substrate steel DD13 and powder AISI 304 (MS—manufacturer specification, EDS—receiving inspection by means of scanning electron microscopy with energy-dispersive X-ray spectroscopy).

Element [wt %]	Fe	Cr	Ni	Mn	Si	Co
AISI 304 (MS)	bal.	19.10	10.60	1.50	0.60	0.17
AISI 304 (EDS)	bal.	19.70	10.50	1.60	0.60	0.20
DD13 (MS)	bal.	0.02	0.02	0.33	0.01	-

## Data Availability

The data presented in this study are available on request from the corresponding author.
